# Editorial: Intrauterine nutrition and adult metabolism

**DOI:** 10.3389/fnut.2022.1085083

**Published:** 2022-11-17

**Authors:** Xinhua Xiao, Lei Su

**Affiliations:** ^1^Key Laboratory of Endocrinology, Ministry of Health, Department of Endocrinology, Peking Union Medical College Hospital, Peking Union Medical College, Chinese Academy of Medical Sciences, Beijing, China; ^2^Department of Geriatrics, First Affiliated Hospital of Sun Yat-sen University, Guangzhou, Guangdong, China

**Keywords:** intrauterine nutrition, maternal malnutrition, offspring, developmental programming, glucolipid metabolism, DOHaD

## Introduction

Intrauterine life is a critical window of development, where a complex interplay between genetics and environmental factors, particularly nutrition, play a central role in defining this transformation and its long-term impact on the rest of an individual's life. Beginning with the “developmental origins of health and disease” (DOHaD) theory put forward by Barker in 1989 (1), extensive epidemiological investigations and experimental animal models demonstrated that intrauterine malnutrition (undernutrition and overnutrition) has profound effects on vulnerability to disease in adulthood (2–9). The fetus adjusts its homeostasis system by making predictive adaptations to the intrauterine environment to help instant survival and to improve later survival in an expected postnatal environment. However, inappropriate predictions of these changes to the intrauterine nutrition environment may lead to a mismatch between nutrition in uterine and postnatal reality, and finally result in an increased risk of chronic diseases in adulthood, which can be transmitted to future generations.

This Research Topic “*Intrauterine Nutrition and Adult Metabolism*” in *Frontiers in Nutrition* collected 5 scientific contributions from highly qualified research groups focusing on early-life nutrition environment and the development of chronic metabolic diseases in later life. Based on these studies, we hope to provide a better understanding of the mechanism and pathogenesis of metabolic disorders in adulthood caused by maternal nutrition in the uterus, as well as potential intervention approaches to interrupt the intergenerational adverse effects.

## Adult metabolic consequences of intrauterine malnutrition

Malnutrition refers to imbalances of energy, protein, and other nutrients. In this regard, Chinese Great Famine (CGF) is a special research model for the study of long-term influence of maternal nutrition deficiencies on the next generation. Since CGF happened three decades ago, it is widely investigated that famine during pregnancy increased the risk of abnormal glucose and lipid metabolism in the offspring (10–12). Interestingly, Zhang et al. provide novel information regarding the sex differences in the long-term effects of CGF. They found prenatal exposure to CGF significantly increased risks of abnormal BMI, blood sugar, triglycerides, and fatty liver in men, whereas women showed increased risks of abnormal blood sugar and positive urine sugar. These results provide new clues in considering different strategies of precise medicine for each sex in early prevention of metabolic dysfunction. Although population-based intrauterine growth restriction (IUGR) has identified a series of associated postnatal complications, whether such metabolic reprogramming remains long-term and whether these alterations could be reversed are currently unknown. Li et al. established an IUGR rat model by a low protein diet. They presented dynamic gene expression changes in offspring cardiac and skeletal muscle among various developmental processes. Although the functional validation was still required, their results have suggested that only improving nutrition postnatally would not completely reverse the damaging effects of IUGR on long-term cardiovascular function. In view of animal studies are essential for understanding the deeply molecular mechanisms that underly DOHaD, other causes induced IUGR models are also valuable for future investigation.

As another side of malnutrition, intrauterine over nutrition (such as gestational diabetes mellitus and obesity) has also been accepted as a detrimental factor on offspring metabolism. The intestinal microbiome is a unique ecosystem that can influence host energy and metabolic homeostasis. The gut ecosystem is thought to be established at or soon after birth and facilitated by vertical transmission and exposure to and/or ingestion of environmental flora (13, 14). Thus, the influences of maternal nutrition status on the offspring's microbiome are significant and may reprogram the disease trajectory. Zheng et al. show maternal high-fat diet (HFD) can reduce microbiota diversity and increase *Lactobacillus* abundance in offspring at weaning and adulthood. It is interesting that the number of differential microbiota species in offspring was decreased in adulthood compare with weaning. Bacterial intervention experiments on offspring will be needed to establish a causal effect. The effects of dietary interventions against malnutrition are also explored. Satokar et al. reported that n-3 PUFA was beneficial to the offspring if the mother consumed an HFD, but deleterious if the mother consumed a control diet. Future work is required to explore the underlying mechanism. The interplay between placenta transport and various metabolic organs of the offspring needs to be further explored.

In addition to metabolic diseases, over nutrition also links to higher risk of cancers (15). In a case-control study, Jonoush et al. examined the relationship between different types of dietary carbohydrates and colorectal cancer (CRC). The authors found a positive association between CRC and dietary intake of carbohydrates, sugar, fructose, sucrose, and maltose, implying the dietary pattern of low-carbohydrate and low-sugar may be an effective intervention for CRC prevention. However, a deep explore of the underlying mechanism and the longitudinal studies of findings verification are needed in the further studies.

## Conclusions and perspectives

There is now compelling evidence for the transmission of poor metabolic health across generations due to adverse intrauterine exposure. Mounting data indicated that *in utero* malnutrition can have an impact on offspring epigenetic profile, microbiome colonization, and mitochondrial function in a manner that is stable postnatally, into adulthood, in association with changed metabolic phenotype. However, just like research progresses in other fields, many unsolved questions remain. Limited evidence exists for a causal role for identified mechanisms in mediating the effects of adverse maternal nutritional conditions on offspring metabolic health. More work should be done to establish longitudinal human studies to build this evidence base, supplemented with ongoing animal model studies that allow direct assessments of relevance. Another important challenge is to reveal the full pathway from maternal exposure to altered structure/function of reproductive organs, and eventually determine offspring later health. For example, Whether the effects of maternal exposure can be reversed in offspring? What are the epigenetic mechanisms, including DNA methylation, histone modifications, and small RNAs transmitted from the mother *via* the oocyte? Can this information be propagated to the next generation *via* the germline?

In summary, this Research Topic provides new insights into the study of intrauterine nutrition and adult metabolism. As have mentioned above, we are still at the very beginning to understand how to prevent the detrimental program effect transform across the generations and protect offspring from metabolic risk. This Research Topic provides a good beginning in this field to find predictive early-life biomarker and enabling targeting of novel interventions for the prevention and control of chronic diseases.

## Author contributions

All authors listed have made a substantial, direct, and intellectual contribution to the work and approved it for publication.

## Conflict of interest

The authors declare that the research was conducted in the absence of any commercial or financial relationships that could be construed as a potential conflict of interest.

## Publisher's note

All claims expressed in this article are solely those of the authors and do not necessarily represent those of their affiliated organizations, or those of the publisher, the editors and the reviewers. Any product that may be evaluated in this article, or claim that may be made by its manufacturer, is not guaranteed or endorsed by the publisher.
